# Assessment of Noise Exposure and Its Characteristics in the Intensive Care Unit of a Tertiary Hospital

**DOI:** 10.3390/ijerph17134670

**Published:** 2020-06-29

**Authors:** Seungho Jung, Jeongmin Kim, Jiho Lee, Chooljae Rhee, Sungwon Na, Jin-Ha Yoon

**Affiliations:** 1 Department of Anesthesiology and Pain Medicine, Severance Hospital, Yonsei University College of Medicine, Yonsei University Health System, Seoul 03722, Korea,; Jungshme@yuhs.ac (S.J.); Anesjeongmin@yuhs.ac (J.K.); 2 Department of Occupational and Environmental Medicine, Ulsan University Hospital, University of Ulsan College of Medicine, Ulsan 44033, Korea; leejh@uuh.ulsan.kr; 3 Asia Noise & Vibration Institute Co., Ltd., Seoul 05641, Korea; goanvi100@anvi.co.kr; 4 Department of Preventive Medicine, Yonsei University College of Medicine, Seoul 03722, Korea; 5 The Institute for Occupational Health, Yonsei University College of Medicine, Seoul 03722, Korea

**Keywords:** intensive care unit, critical care, facility design and construction, noise

## Abstract

Noise generated in the intensive care unit (ICU) adversely affects both critically ill patients and medical staff. Recently, several attempts have been made to reduce ICU noise levels, but reliable and effective solutions remain elusive. This study aimed to provide evidence on noise distributions in the ICU to protect patient health. For one week, we measured noise levels in isolated rooms, open units, and nursing stations in medical, surgical, and pediatric ICUs, respectively. We additionally analyzed the noise generated by medical equipment that is frequently used in ICUs. The median (interquartile range) noise exposure level (dBA) of all ICU units was 54.4 dB (51.1–57.5) over 24 h. The highest noise exposure was noted in the surgical ICU’s daytime open unit at 57.6 dB (55.0–61.1). Various ICU medical devices continuously generated low-frequency noise. Mechanical noise levels ranged from a minimum of 41 dB to a maximum of 91 dB. It was also confirmed that patient-monitoring devices generated loud, high-frequency noise at 85 dB. ICU noise levels were much higher than expected. Noise reduction that focuses on behavior modification of medical staff has limited potential; instead, structural improvements should be considered to reduce the transmission of noise.

## 1. Introduction

The hospital intensive care unit (ICU) is an important facility designed to provide critical patients with specialized care and close monitoring of vital signs. Every step of ICU work is focused on patient care, but the required activities and medical devices generate noise [1]. Noise is a well-known environmental hazard that can lead to adverse auditory and nonauditory health effects such as sleep disturbances, cardiovascular effects, and psychological problems [2]. The World Health Organization (WHO) guidelines suggest that the A-weighted energy-equivalent sound pressure levels (SPLs) (in LAeq) and A-weighted maximum SPLs with fast time constants (in LAmax) in hospital ward rooms should be 30 and 40 dB, respectively [3]. Furthermore, the WHO suggests that hospital treatment rooms should keep noise levels “as low as possible” to prevent interference with rest and recovery [3]. Noise-induced stress and hyperarousal [4] can also disrupt workers’ health and job performance, resulting in unexpected hazardous situations for ICU patients. 

Recent research has reported that an LAeq of 57 dB and LAmax of 83 dB have been observed in neonatal intensive care units (NICU). These noise exposures are almost two times higher than those recommended by the WHO [5]. According to the WHO, evening and nighttime noise exposure should be 5 to 10 dB lower than daytime noise exposure. However, in the NICU, the noise exposure was not well attenuated in the evening and at night [5]. Hence, there have been interventions to reduce noise exposure levels in hospitals. A quiet bundle team approach with education and behavioral change reduced noise exposure levels by an average of 3 dB, with patients and health care workers reporting decreased perceptions of noise [6]. The “quiet times” approach also reduced noise exposure levels by an average of 3 dB through participatory programs that involved education, the control of equipment, and behavioral and environmental modifications [5]. Overnight “quiet time” intervention programs in the surgical ICU (SICU) attenuated noise exposure from 62.5 dB to 59.6 dB, and the attenuation effects were more pronounced near patient areas [7]. Although various approaches to noise reduction have been moderately successful [1,5,6,7], the levels of reduction they achieved were not sufficient to protect patient health. The difficulty in achieving noise reduction has been a lack of evidence about modifiable noise sources. For this reason, the use of comprehensive data sources would be helpful to determine the “what, where, and when” of noise in the ICU setting. 

A comprehensive analysis of noise exposure distribution is therefore required to obtain evidence regarding noise reduction in ICU settings. The current study aimed to provide evidence on noise distributions in the ICU in the interest of protecting patient health. To accomplish this, we measured noise levels over specific time intervals, including days, evenings, nights, weekdays, and weekends, in settings such as medical, surgical, and pediatric ICUs (M-, S-, and P-ICU, respectively). 

## 2. Materials and Methods 

This study was performed at Severance Hospital (Seoul, Korea) from 5 November, 2018 to 12 November, 2018. This study was conducted in accordance with the principles of good clinical practice and was approved by the Institutional Research Board of Severance Hospital (reference number 4–2018–0366). 

### 2.1. SPL Measurements 

**Figure 1:**
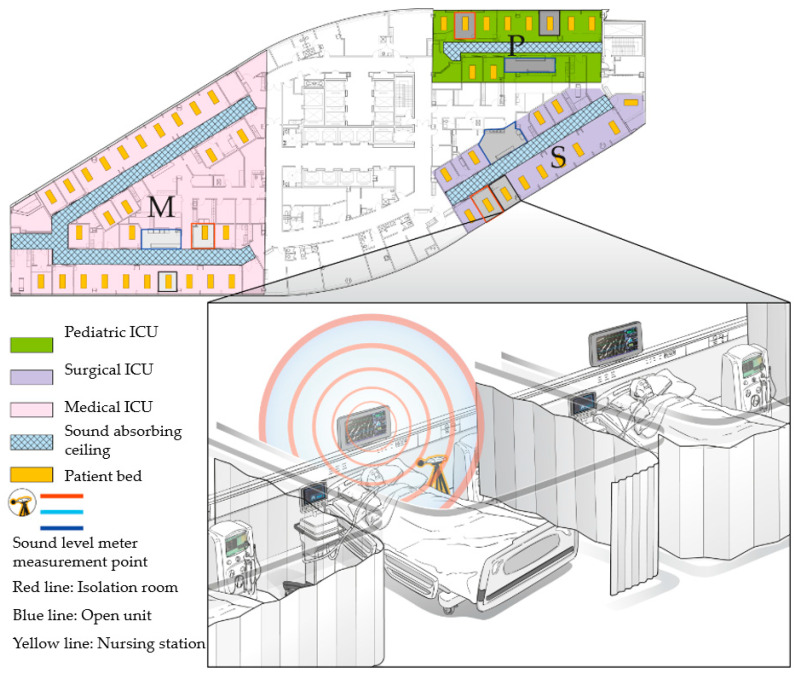
Map of the intensive care unit (ICU) and sound level meter measurement points. The sound pressure level (SPL) measurements were continuous at three points: band during the day (06:00–14:00), evening (14:00–22:00), and night (22:00–06:00), according to the three work shifts of the nurses. The sound levels were recorded every second over eight days at each location.

All noise measurements were obtained through microphones that convert acoustic energy to electrical energy for further analysis. These microphones measure sound using a precise sound level meter and an A-weighting filter to simulate the frequency response of human ears. The sound level meters were positioned at three different locations: on the ceilings above the nursing station (NS) desk; near the heads of beds (within 30 cm) in isolated rooms (IRs); and in open units (OUs) (Figure 1). 

### 2.2. SPL Analysis and Evaluation Method

The overall time histories of noise exposures in the ICU were statistically analyzed to determine their maximum, minimum, and mean values. The sound levels were classified as less than 30 dBA, less than 40 dBA, less than 50 dBA, less than 60 dBA, and over 60 dBA by accumulating the duration times for days, evenings, and nights. Additionally, the magnitude and frequency of noise generated by medical devices frequently used in the ICU were analyzed, and the maximum and minimum values were measured in the IRs of each ICU.

**Figure 2:**
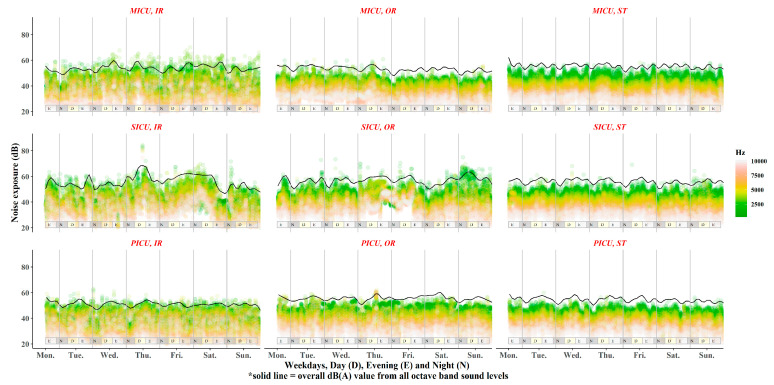
Noise exposure time trends of individual and overall dBA values for octave band sound levels

The noise exposures over time in all octave bands and the overall SPL (dBA) sound levels were plotted. Differences in noise exposures between weekdays and weekends were assessed. To obtain *p*-values, a t-test and Wilcoxon rank test were performed, and *p*-values below 0.05 were considered statistically significant. Overall SPL ($L^{\prime }$) was estimated by the following formula: $L^{\prime }$ = $10\times \sum^{\text{​}}\log\left(10^{\frac{L_{n}}{10}}\right)\;$ [dB]. In the graph, the overall SPLs were plotted with the smoothing function of LOWESS (Figure 2). 

The differences in noise exposure levels were subjected to a two-sided t-test and a Wilcoxon rank sum test, and *p*-values below 0.05 were considered statistically significant. All statistics were performed with R statistics. 

## 3. Results

The median (interquartile range (IQR)) noise exposure level (dBA) for all ICU units during the study period was 54.4 (51.1–57.5) over a 24 h period, with no difference between day and night. The SICU, which had a higher frequency of patient admission and discharge, had higher noise levels than either the MICU or PICU. During the nights, when patients need to sleep and rest, the SICU reported the highest noise exposure level, at 53.4 (50.5–56.6) dB (Table 1).

The SICU also had higher rates of noise exposure over 60 dB than other intensive care units. In particular, we observed that the SICU’s OU was exposed to more than 60 dB of noise for 19.1% of the total measurement time in the evening (Table 2).

In Figure 2, the level of low-frequency noise (green) is higher than that of other frequencies for all ICUs across all time zones. Fluctuations in SPLs occurred more often at low frequency.

**Figure 3:**
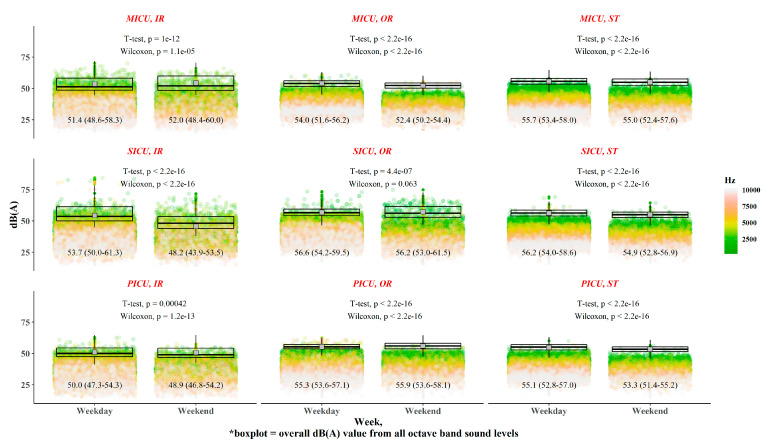
Noise exposure levels on weekdays versus weekends. Note: The boxplot indicates differences in overall sound pressure level (dBA) values for all octave band sound levels.

Generally, the noise exposure level on weekdays was higher than that on weekends (Figure 3). However, the noise exposure in the OU of the SICU was not attenuated on weekends compared with that on weekdays (*p* = 0.063 by Wilcoxon rank sum test). 

Table 3 shows the wave patterns and magnitudes of noise produced by frequently used medical devices. There are three types of alarms on the patient main monitor (Table 3). First, the inoperative (INOP) alarm indicates that a monitor is not working and is often called the blue alarm. If the monitor does not recognize or measure the patient’s condition, this information is displayed in blue on the screen and a low-frequency noise is generated. The second alarm is yellow and has a higher frequency SPL than the blue alarm. The yellow alarm is a saw-type alarm that automatically stops within 6 s if physiologic parameters return to normal values. The red alarm has a flat wave and a high pitch, and the SPL goes up to 85 dB. This alarm is for critical situations, and it does not stop unless the medical staff turns it off after checking the value on the screen. In addition to the alarm systems, many devices used in daily routine patient care continuously produce low-frequency noises (Table 3). 

## 4. Discussion

This study verified that ICUs are very noisy areas and that noises occur constantly at night and during the day. The SPL level at night was 52.7 dB on average—much higher than the night noise level of 30 dBA recommended by the WHO. Furthermore, this level was higher than the 42 dBA noise level at which sleep quality is affected. Interestingly, although the researchers assumed that the IR would be quieter than other locations, in reality the percent distribution of noise exposure levels greater than 60 dBA was higher in the IR than in the OU of the MICU at night (6.4% vs. 2.7%, respectively). 

In this study, the low-frequency noise levels showed higher SPLs than other frequency levels for all ICUs across all time zones. The low-frequency noise SPLs were continuously produced by ventilators, continuous hemodialysis machines, respiratory physiotherapy machines, and intermittent pneumatic compression devices throughout the day. These repetitive, low-frequency machine sounds are produced by various medical devices essential to ICU care, resulting in unavoidable noise 24 h a day.

Our findings revealed that the noise level in the SICU was higher than that in the MICU or PICU. In particular, the nocturnal noise level in the SICU was 53.4 dB. A possible explanation for this is that postsurgical patients are admitted to the SICU late in the afternoon or even at night. Immediately following ICU admission, several assessments are performed: meticulous fluid management, careful transfusion, mechanical ventilator setting, proper pain control, adequate sedation, and surgical site close observation (for postoperative patients). These assessments usually take 10–30 min. Moreover, 3–4 health care workers (e.g., intensivists and nurses) are needed in the early stages of ICU admission, and they inevitably generate noise. Therefore, the noise generated at this stage is difficult to avoid, and to some extent, the SICU medical staff takes this for granted. However, it is clear that nocturnal noise detracts from the sleep quality of ICU patients, particularly since ICU patients are hemodynamically very unstable and are attached to multiple monitoring devices. Each device has an alarm system, which sends out continuous auditory signals that can be heard by the surrounding patients. Recent studies have suggested that alarms in ICUs cause noise [8] and that this noise can be reduced with alarm modifications [9]. It would be useful to improve alarm systems such that errors and extraneous alarm noise could be reduced, but if the alarm frequencies were to decrease, the medical staff might become anxious about whether alarms were functioning properly. Therefore, a more realistic and practical solution would be to accept that some noise in the ICU is inevitable and focus on reducing the adverse effects on the surrounding patients.

Noise reduction can be approached from two directions: reducing loud noises over 60 dB and reducing continuous noises over 45 dB. Table 2 shows that in the NS, noises of more than 60 dB occurred during 8.6% of the total measurement period. In the NS, noise sources include nursing-related conversations or phone calls, computers, and the alarm system for the central monitoring of all ICU patients. To reduce these noise sources, sound-proofing the walls and ceilings, installing computer booths, and implementing visual warning systems at nursing stations have been recommended [10,11]. Expanding the sound-absorbing ceilings present in the corridors to the NS would by itself produce an expected reduction in the average noise of 3 dB [10]. 

At all of the measurement points (IR, OU, NS), noise greater than 45 dB occurred during >90% of the measurement period, regardless of whether it was day or night. A solution to this problem would be to reduce the noise from the continuous use of medical equipment as much as possible by employing “matching control techniques.” For example, the high-frequency noises from obstruction during suction catheter use can be reduced up to 10 dB by switching to a catheter tip that is less stiff and less prone to clogging and by changing to a noise absorption hose. Low-frequency mechanical noises such as those from hemodialysis machines can be reduced with sound-absorbing panels or trellises [12]. In addition, using rubber wheels on mobile devices can minimize the noise from friction between the wheels and the floor [13]. Using silicone covers to reduce mechanical vibration can also reduce noise, and removing medical machines from walls can also reduce noises that are caused by sound reflection. Therefore, the design of hospital rooms should be carefully reviewed and should incorporate factors beyond just infection control measures. These should include consideration of noise exposure to optimize the quality of patient care. 

In 2017, the Korean Ministry of Health and Welfare announced a bill that would strengthen the standards for hospital rooms, such as the distance between beds. The new standards for medical facilities require drastic revisions to the medical environment to enhance its overall features and to supply items to improve patient safety and the quality of patient care. For example, ICU isolation rooms must have an area of at least 15 m^2^, the distance between the wall and the bed should be at least 1.2 m, and the distance between beds in open rooms should be at least 2 m. However, these revisions do not include strategies for qualitative improvements to treatment spaces that affect the ambience of patient rooms. For example, qualitative improvements that have not been mentioned but could be considered include maintaining certain light intensities in rooms to help regulate patients’ circadian rhythms, installing sound-absorbing panels to reduce environmental noise, using antimicrobial building materials to reduce nosocomial infections, and installing high-efficiency particulate air filters to guarantee air quality. Therefore, the noise exposure status findings described in this study suggest that a change in medical environmental policy is required to improve patient health. Both quantitative and qualitative criteria for noise levels should be considered when establishing these practical policies.

There is growing evidence that the sleep quality of ICU patients is closely tied to their recoveries, and it has been repeatedly emphasized that nocturnal noise in ICUs should be reduced so that patients’ sleep quality can be improved [14]. There have been many studies on the causes of ICU noise and ways to reduce it [15,16,17]. Many observational studies have been conducted on the adverse effects of noise, but there have been very few clinical studies on how noise affects the recovery of critically ill patients. This is due to the fact that, when treating critically ill patients, the highest priority has been intensively treating patients rather than improving their surrounding environment. It is now necessary to develop practical strategies for changing the structure of hospital environments rather than the behavior of medical staff. In this regard, our study is meaningful in that it evaluates the real conditions of various types of ICUs and recommends practical improvements. 

Our research has a definite limitation due to being a single-center observational study. However, our findings shed light on the noise characteristics of each specialized ICU. In this study, we suggest that understanding the noise characteristics of each ICU and modifying ICU structures would help to reduce the actual noise generation. For example, if a patient in an SICU is undergoing a procedure, a temporary sound-absorbing panel could be installed around the patient to reduce noise. Furthermore, although regional noise assessments were undertaken, actual noise exposure from the individual modifiable environment was not. Therefore, more individualized assessments of noise exposure will be required to make preventive plans that are both efficient and effective.

## 5. Conclusions

The noise levels in the ICU were much higher than expected. Depending on the type of ICU, the frequency of noise generation may vary, but the reality is that intensive care is performed 24 h per day, 7 days per week. Thus, noise is generated throughout the day. Based on noise characteristics in the ICU, this study recommends attenuating noise through cost-effective interventions that require minimal effort to deploy, such as replacing textile curtains with functional sound-absorbing ones. When designing ICUs, soundproofing strategies should be seriously considered.

## Figures and Tables

**Table 1:** Median (IQR) noise exposure levels (dBA) according to each ICU and time schedule.

		24 h	Day	Evening	Night
**MICU**	IR	51.6 (48.5–58.9)	53.2 (49.5–59.1)	52.5 (48.9–59.8)	49.3 (47.8–56.9)
	OU	53.2 (51.0–55.4)	53.8 (51.8–55.9)	53.8 (51.9–55.9)	51.5 (49.7–53.8)
	NS	55.4 (52.9–57.7)	56.0 (53.5–58.1)	55.6 (53.6–57.8)	54.4 (51.6–57.0)
	Total	53.8 (50.6–57.0)	54.6 (51.6–57.4)	54.3 (51.5–57.4)	52.0 (49.2–55.9)
**SICU**	IR	51.9 (47.5–58.0)	53.4 (48.6–61.9)	51.3 (47.4–56.6)	51.2 (47.1–56.1)
	OU	56.5 (53.9–59.9)	57.6 (55.0–61.1)	57.3 (54.7–60.2)	54.4 (51.7–57.1)
	NS	56.0 (53.8–58.4)	56.9 (55.2–59.2)	56.6 (54.6–58.8)	53.9 (52.0–56.3)
	Total	55.4 (52.0–58.8)	56.7 (53.6–60.2)	55.9 (52.8–59.0)	53.4 (50.5–56.6)
**PICU**	IR	49.7 (47.1–54.3)	50.5 (48.0–55.3)	50.5 (47.8–55.2)	47.8 (45.5–52.0)
	OU	55.4 (53.6–57.4)	56.0 (54.0–58.1)	55.9 (54.3–57.6)	54.2 (52.3–56.4)
	NS	54.6 (52.2–56.6)	55.5 (53.8–57.3)	54.7 (52.3–56.9)	53.0 (50.9–55.2)
	Total	54.1 (50.7–56.7)	54.9 (52.1–57.2)	54.5 (51.2–57.0)	52.6 (49.1–55.3)
**Total**	IR	51.0 (47.8–57.1)	52.4 (48.7–58.2)	51.2 (48.2–57.4)	49.4 (47.0–54.6)
	OU	54.9 (52.6–57.5)	55.8 (53.5–58.3)	55.6 (53.5–57.9)	53.5 (51.0–56.0)
	NS	55.3 (52.9–57.6)	56.1 (54.2–58.2)	55.7 (53.5–57.9)	53.7 (51.5–56.2)
	Total	54.4 (51.1–57.5)	55.4 (52.3–58.2)	54.9 (51.7–57.8)	52.7 (49.6–56.0)

Day: 6:00–14:00, Evening: 14:00–22:00, Night: 22:00–6:00; IR: isolation room, OU: open unit, NS: nursing station; MICU: medical intensive care unit, SICU: surgical ICU, PICU: pediatric ICU.

**Table 2:** Noise exposure level distribution seconds (%) according to each ICU and time schedule.

Noise Exposure (Unit: dB)		MICU				SICU				PICU			
		24 h	Day	Evening	Night	24 h	Day	Evening	Night	24 h	Day	Evening	Night
IR	<30	0.00	0.00	0.00	0.00	0.40	2.00	0.10	0.10	0.00	0.00	0.00	0.00
	<40	0.00	0.00	0.00	0.00	0.20	0.00	0.00	0.70	0.00	0.00	0.00	0.00
	<50	69.00	60.70	67.70	78.90	68.10	66.20	73.40	75.80	76.50	73.00	75.40	84.90
	<60	23.60	30.80	24.20	14.70	20.60	23.90	19.30	18.50	19.80	23.00	20.70	12.40
	≥60	7.40	8.50	8.20	6.40	10.70	7.90	7.20	4.90	3.70	4.10	3.90	2.70
OU	<30	0.00	0.00	0.00	0.00	0.00	0.00	0.00	0.00	0.00	0.00	0.00	0.00
	<40	0.10	0.00	0.00	0.20	0.00	0.00	0.00	0.00	0.00	0.00	0.00	0.00
	<50	45.50	40.90	49.10	61.90	27.90	19.90	21.00	41.60	13.50	9.80	11.10	24.00
	<60	49.50	54.20	46.50	35.20	56.90	59.90	59.90	50.20	77.30	79.30	81.20	70.70
	≥60	4.90	4.90	4.40	2.70	15.20	20.20	19.10	8.30	9.30	10.90	7.70	5.30
NS	<30	0.00	0.00	0.00	0.00	0.00	0.00	0.00	0.00	0.00	0.00	0.00	0.00
	<40	0.00	0.00	0.50	0.00	0.00	0.00	0.00	0.00	0.00	0.00	0.00	0.00
	<50	41.40	34.70	44.60	53.10	36.80	22.50	31.80	53.10	44.30	32.60	49.50	59.90
	<60	50.10	54.80	46.80	39.80	53.80	63.30	58.90	40.90	49.40	59.50	45.40	36.10
	≥60	8.60	10.40	8.10	7.20	9.40	14.10	9.30	6.00	6.30	7.80	5.10	4.00

Day: 6:00–14:00, Evening: 14:00–22:00, Night: 22:00–6:00; IR: isolation room, OU: open unit, NS: nursing station; MICU: medical intensive care unit, SICU: surgical ICU, PICU: pediatric ICU.

**Table 3:** Wave pattern analysis of noise generated by various medical devices in the ICU.

Medical Device	Type of Noise	Peak Hz	SPL Range (Min–Max)	SPL Average (Leq)	Pattern Type
Patient monitor (Philips IntelliVue)	INOP alarm	160	42.9 67.5	62.8	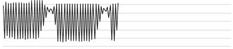
	Yellow alarm	160	45.5 69.3	63.5	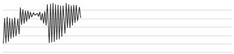
	Red alarm	630	76.0 84.9	80.7	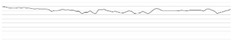
Oxygen supply via facial tent mask	Low flow (60%, 5 L/min)	400	63.2 66.0	64.3	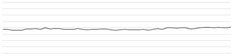
	High flow (80%, 10 L/min)	500	69.3 71.4	70.4	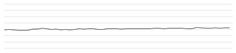
Syringe pump (Terumo^®^)	INOP alarm	250	60.8 68.7	66.8	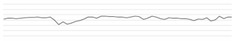
	Yellow alarm	250	55.7 67.8	63.4	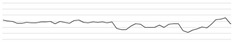
Pneumatic compression device	Normal operation	250	43.9 51.6	46.9	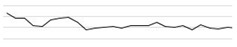
Nebulizer	Ventilator connection	200	48.5 70.9	58.1	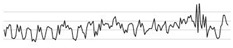
Endotracheal suction	Preparation & procedure	200	57.2 67.2	59.5	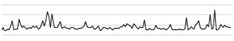
Wall suction	Normal operation	500	58.1 59.9	58.7	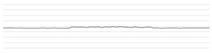
	Obstruction	500	78.2 91.6	86.4	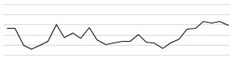
Warmer	Bair warmer	200	55.1 61.5	57.0	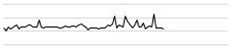
Ventilator	Alarm	500	78.6 82.7	80.7	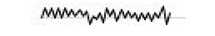
	Normal operation	160	48.0 53.3	50.2	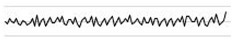
Respiratory physiotherapy	Normal operation	200	64.9 67.5	67.1	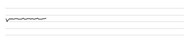
Continuous renal replacement therapy	INOP alarm	400	49.1 63.0	58.3	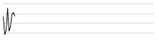
	Yellow alarm	315	47.9 62.4	58.0	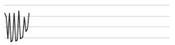

INOP alarm, inoperative alarm.
